# Supplementary material for: Hand Hygiene during the Early Neonatal Period: A Mixed-Methods Observational Study in Healthcare Facilities and Households in Rural Cambodia
Source: Int J Environ Res Public Health. 2021 Apr 21;18(9):4416. doi: 10.3390/ijerph18094416 (PMC8122667; doi:10.3390/ijerph18094416)
Supplement: Supplementary file 1 [file ijerph-18-04416-s001.zip › ijerph-1170257-supplementary.pdf]

Table S1. Adapted Behaviour Centred Design (BCD) checklist of handwashing behavioural components and determinants.

BCD Component	Determinant	Definition of Each Determinant Adapted to Handwashing
**Brain**	Executive Brain	• The extent to which knowledge of handwashing behaviour and its benefits affects handwashing intentions and plans, and eventually performance of the behaviour.
	Motivated Brain	• The goal-related drivers of behaviour. Motives for handwashing can include (but are not limited to) disgust (the desire to avoid cues to sources of infection), affiliation (the desire to fit in with others) and nurture (the desire to care for your child).
	Reactive Brain	• The extent to which handwashing can be automatically triggered based on past experience and repetition.
	Discounts	• The perceived time, effort and costs of washing hands with soap as compared to other courses of action.
**Body**	Characteristics	• Socio-demographic characteristics that may affect handwashing, including gender, wealth, age, education and employment.
	Senses	• The sensory perceptions that may cue handwashing behaviour or be experienced during or after handwashing.
	Capabilities	• Whether an individual has the skills required to wash their hands with soap.<br>• Whether an individual perceives themselves to be able and willing to actually wash their hands at the times required.
**Behaviour settings**	Stage	• The design and set up of the specific physical spaces where handwashing behaviour takes place.
	Infrastructure	• Durable infrastructure associated with handwashing such as water supply systems, sanitation, kitchen facilities and handwashing facilities.
	Props	• The value, characteristics, usability, ownership and accessibility of soap and other objects used for handwashing.
	Roles	• The ways in which an individual's role, identity or responsibilities influence their handwashing practices.
	Routine	• The sequence of behaviours regularly performed in association with handwashing.
	Norms	• The extent to which an individual's handwashing practice is influenced by their perception of normative setting-specific rules. This includes an individual's perception of whether handwashing is commonly practiced in their community (descriptive norm); whether handwashing is part of their role and their normal behaviour (personal norm); whether handwashing is socially approved of (injunctive norm); and whether handwashing is practiced by their 'valued others' (subjective norm).
**Environment**	Physical environment	• Factors in the physical or built environment including climate and geography.
	Biological Environment	• Factors associated with an individual's interaction within their biological environment.

	Social Environment	• The structure of an individual's social environment, including how they interact with it and perceive themselves within it.
**External context**	Political and historical context	• The historical and cultural events that have shaped current perceptions and practices of handwashing. The extent to which handwashing-related policies or local and national leadership on handwashing issues, shape handwashing perceptions and practices at the individual level.
